# Protective effects of *Tinospora crispa* and *Tinospora cordifolia* extracts against methylglyoxal-induced oxidative stress and apoptosis in hFOB 1.19 cells: ELISA-based evaluation and molecular docking insights with antioxidant enzymes

**DOI:** 10.1186/s12906-026-05450-y

**Published:** 2026-06-24

**Authors:** Luqman Jaya, Gabriele Ruth Anisah Froemming, Zunika Amit

**Affiliations:** 1 Faculty of Medicine and Health Sciences, Universiti Malaysia Sarawak, Kota Samarahan, Sarawak 94300 Malaysia; 2 Centre of Preclinical Sciences, Faculty of Dentistry, Universiti Teknologi MARA, Sungai Buloh, 47000 Selangor Malaysia

**Keywords:** Molecular docking, Osteoblast, Oxidative stress, *Tinospora cordifolia*, *Tinospora crispa*

## Abstract

**Background:**

Excessive production of reactive oxygen species (ROS) and advanced glycation end products (AGE) induced by methylglyoxal (MGO) contribute to oxidative stress and cellular apoptosis in osteoblasts, ultimately disrupting bone homeostasis. This study aimed to investigate the protective effects of *Tinospora crispa* and *Tinospora cordifolia* extracts against MGO-induced oxidative stress and apoptosis in hFOB 1.19 osteoblastic cells.

**Methods:**

Methanolic and aqueous extracts were evaluated for their ability to reduce intracellular ROS, caspase-3 expression, and AGE expression through ELISA based assays. Molecular docking was performed between selected bioactive compounds of these extracts and related proteins through AutoDock Vina. Statistical analyses were performed using ANOVA, followed by post-hoc tests.

**Results:**

Methanolic extracts of *T. crispa* (240 µg/mL) and *T. cordifolia* (100 µg/mL) significantly reduced intracellular ROS levels by 46.8% and 41.2%, respectively (*p* < 0.05). Caspase-3 expression in MGO-induced osteoblast cells was significantly decreased following treatment with *T. crispa* and *T. cordifolia* extracts, indicating anti-apoptotic effects. However, only methanolic *T. cordifolia* (100 µg/mL) and aqueous *T. crispa* at (375 µg/mL) significantly reduced intracellular AGE expression (*p* < 0.05), suggesting selective effects on oxidative stress pathways. Molecular docking analysis revealed strong binding affinities of key bioactive compounds particularly towards berberine with xanthine oxidase (-8.3 kcal/mol) and caspase-9 (-8.7 kcal/mol). Meanwhile, superoxide dismutase showed a strong binding affinity towards quercetin (-7.9 kcal/mol).

**Conclusion:**

These findings suggest that *T. crispa* and *T. cordifolia* extracts possess significant antioxidant and anti-apoptotic properties, making them promising therapeutic agents in mitigating MGO-induced osteoblastic damage.

**Supplementary Information:**

The online version contains supplementary material available at https://doi.org/10.1186/s12906-026-05450-y.

## Background

Sustained elevation of reactive oxygen species (ROS) serves as a primary cause of systemic cellular dysfunction [1]. This pathological oxidative surge is predominantly initiated through the accumulation of methylglyoxal (MGO), a highly reactive α-dicarbonyl metabolite [2, 3]. As a potent glycating agent, MGO acts as a primary chemical precursor that undergoes non-enzymatic reactions with cellular proteins, leading to the advanced glycation end-products (AGE) [4]. The combined accumulation of MGO and AGE enhances intracellular ROS production, thereby intensifying cellular stress which leads to apoptosis [5].

Within the skeletal system, persistent oxidative stress directly disrupts bone turnover homeostasis, significantly elevating the risk of diabetic bone dysfunction [6]. Under healthy physiological conditions, the bone turnover rate is balanced, maintaining an equilibrium between osteoblasts that synthesise new bone matrix and osteoclasts that resorb ageing bone [7]. The resulting oxidative stress environment triggers a potent apoptotic cascade within these bone-forming cells, culminating in accelerated osteoblast mortality. In various in vitro models of oxidative stress, cellular apoptosis is consistently characterised by high expression of caspase-3, the primary executioner protease of programmed cell death [8]. Consequently, the loss of viable osteoblasts interrupts the delicate balance of bone remodelling, causing disruptions of skeletal system’s bone formation-resorption equilibrium. To counteract this degenerative shift, natural therapeutic interventions are increasingly being explored, with specific attention given to *Tinospora crispa* (*T.* crispa) and *Tinospora cordifolia* (*T.* cordifolia) extracts. In previous studies, their activity has been demonstrated using DPPH and FRAP assays [9] and is further supported by both in vitro and in vivo studies demonstrating antioxidant and anti-inflammatory effects [10, 11]. *T. cordifolia* (Guduchi) is widely used in Ayurvedic medicine [12], whereas *T. crispa* (Makabuhay) is commonly utilised in Southeast Asian traditional medicine for treating fever, inflammation, and oxidative stress-related conditions [13]. Furthermore, phytochemical analyses have identified key bioactive compounds in both species, including alkaloids (berberine, palmatine) and flavonoids (apigenin, quercetin), which are well recognised for their antioxidant properties [14–16].

Given that progressive skeletal degradation is heavily driven by oxidative stress and accelerated cell death, identifying the specific molecular targets of these botanicals is crucial. Consequently, contemporary pharmacological studies frequently employ in silico molecular docking to predict the binding affinity and inhibitory potential of plant-derived ligands against key enzymatic regulators such as xanthine oxidase (XO) and superoxide dismutase (SOD), as well as the intrinsic apoptotic initiator, caspase-9.

XO plays a central role in ROS generation and contributes to oxidative stress associated with MGO exposure [17] by producing reactive species such as superoxide and hydrogen peroxide [18]. Excessive XO activity promotes ROS accumulation, which may contribute to oxidative damage and activation of apoptotic pathways in osteoblasts [19]. Accordingly, disrupting XO has been associated with reduced intracellular ROS levels and improved cellular redox balance [20]. Besides, SOD serves as a primary antioxidant defence by catalysing the dismutation of superoxide radicals into oxygen and hydrogen peroxide. In MGO-induced cellular damage, SOD is critical for limiting ROS overproduction and mitigating AGE-associated cellular damage, both of which contribute to cellular dysfunction and apoptosis [21, 22]. Predicting the binding affinity toward SOD provides crucial insights into how these compounds might modulate cellular responses to oxidative stress, highlighting its importance as a key mechanistic target in the study. Finally, caspase-9 is an initiator of the intrinsic apoptotic pathway which can be activated under conditions of oxidative stress and mitochondrial dysfunction [23]. Hence, predicting the competitive binding or potential inhibition of caspase-9 suggests a mechanism by which these compounds could interrupt the apoptotic cascade and mitigate MGO-induced cellular damage.

Despite the recognised medicinal properties of *T. crispa* and *T. cordifolia*, their precise molecular mechanisms in protecting osteoblasts from oxidative stress and apoptosis remain insufficiently defined. Given that MGO-induced cellular damage involves interconnected pathways of oxidative stress, glycation, and apoptosis, targeting key regulators such as XO, SOD, and caspase-9 may provide a mechanistic basis for reducing ROS production, modulating apoptotic signalling, and limiting AGE-associated cellular damage. Therefore, this study evaluates the protective effects of *T. crispa* and *T. cordifolia* extracts against MGO-induced hFOB 1.19 osteoblastic cells. Specifically, intracellular ROS levels, caspase-3 expression, and AGE expression are assessed using ELISA-based assays. Furthermore, molecular docking is performed to predict interactions between selected bioactive compounds and key target proteins, including XO, SOD, and caspase-9. This integrated approach provides critical insights into the protective roles of these extracts in preserving osteoblast function under oxidative stress conditions.

## Methods

### Plant extracts preparation

The powdered leaves and stems of *T. crispa* were purchased from Shine Tech Solutions, Pahang, Malaysia. Meanwhile, powdered leaves and stems of *T. cordifolia* were purchased from Gtee Botanical Extract Pvt. Ltd., Chennai, India (ISO 9001 and ISO 22000). Twenty grams of *T. crispa* plant powder and 20 g of *T. cordifolia* powder were separately mixed with 400 mL of 90% methanol (Fisher Scientific, UK). Each mixture was left to sit in the dark at 24–25 °C for 24 h. The mixtures were then filtered through filter paper with a pore size of 8–12 μm (filtraTECH, France). The solvent was subsequently evaporated using a rotary evaporator. The same procedure was applied to prepare boiled water extracts (aqueous extracts) at 100 °C. After rotary evaporation, the extracts were freeze-dried procedure to fully removed excess water before being stored at -20 °C for further use.

### Cell culture of hFOB 1.19

The foetal human osteoblast cell line hFOB 1.19 was purchased from American Type Culture Collection (ATCC, USA). The cells were cultured in 5 mL of Dulbecco’s Modification of Eagle’s Medium (DMEM)/F-12 supplemented with L-glutamine, 15 mM HEPES (Corning, NY, USA), 10% foetal bovine serum (FBS) (Corning, NY, USA) and 1% penicillin–streptomycin (Corning, NY, USA) in T-25 cell culture flasks (Corning, NY, USA). Cells were maintained at 34 °C in a humidified atmosphere containing 5% CO_2_. Passage 7 cells were used for all cell-related assays unless otherwise stated.

###  In vitro study design

The experimental design included several groups to evaluate the effects of the extracts and control treatments. Prior to treatment, the IC₅₀ of MGO was determined in hFOB 1.19 cells. The two highest extract concentrations that maintained > 80% cell viability were selected for further experiments. Upon reaching confluence, the cells were divided into six treatment groups: (1) cells treated with methanolic *T. cordifolia* extract (2), cells treated with methanolic *T. crispa* extract (3), cells treated with aqueous *T. cordifolia* extract (4), cells treated with aqueous *T. crispa* extract (5), MGO-induced control cells, and (6) untreated cells without MGO or extracts. MGO was used to induce oxidative stress in the osteoblast cells. Following preliminary dose-response studies, methanolic *T. crispa* extracts were administered at two concentrations of 120 µg/mL and 240 µg/mL, methanolic *T. cordifolia* at 50 µg/mL and 100 µg/mL, aqueous *T. crispa* at 187.5 µg/mL and 375 µg/mL and aqueous *T. cordifolia* at 62.5 µg/mL and 125 µg/mL respectively. Cells were treated for 24 h after which cell lysates were collected for the measurement of AGE and caspase-3 levels using ELISA based assays.

### Cell lysate preparation

The hFOB 1.19 cells were washed twice with cold Dulbecco’s Phosphate Buffered Saline (DPBS). Then, 1 mL of RIPA lysis buffer (Thermo Fisher Scientific, USA) was added to the flask, which was placed on ice with gentle agitation for 10 min. Cells were scrapped and transferred into labelled microcentrifuge tubes, and centrifuged at 16,000×g for 15 min at 4 °C. The supernatant (lysate) was then collected into fresh tubes and stored at -80 °C until further use in caspase-3 and AGE ELISA analyses.

### Measurement of intracellular ROS

Intracellular ROS levels were measured using Oxiselect™ Intracellular ROS Green Fluorescence Assay Kit (Cell Biolabs, California, USA). hFOB1.9 cells were seeded in triplicate at a density of 2 $\:\times\:$ 10^4^ cells/mL in black opaque 96-well plate and incubated at 34 °C with 5% CO_2_ until confluence. Cells were then treated according to the six treatment groups as mentioned previously for 24 h. After treatment, the media was removed and cells were washed three times with Hanks’ Buffer Salt Solution (HBSS). Subsequently, 100 µL of 1 $\:\times\:$ DCFH-DA/media solution was added to each well and incubated for 60 min. Following incubation, cells were washed three times with again HBSS. Fluorescence intensity, expressed as relative fluorescence unit (RFU) was immediately measured using a microplate reader (Spectramax iD 3) at an excitation of 480 nm and emission wavelength of 530 nm. The RFU of the treated wells were normalised towards untreated wells and calculated accordingly.

### Measurement of caspase-3 expression

Caspase-3 protein expression in hFOB 1.19 was quantified using an ELISA kit (Cusabio, Texas, USA). Briefly, 100 µL of samples were added to each well and incubated for 30 min at 37 °C. Wells were washed three times with 200 µL of 1× wash buffer followerd 100 µL of HRP-conjugate and incubated for further 30 min at 37 °C. After washing five times with 200 µL of 1× wash buffer, 90 µL of TMB-substrate was added and followed by incubation in the dark for 20 min. The reaction was stopped with stop solution, and absorbance was measured at 450 nm.

### Measurement of AGE expression

AGE expression was quantified using an ELISA kit (Cell Biolabs Inc., California, USA). Briefly, 50 µL of sample was added to each well of the AGE conjugate-coated plate and incubated at room temperature for 10 min. Then, 50 µL of diluted anti-AGE antibody was added and incubated for 1 h at 37 °C with gentle agitation. Wells were washed three times with 250 µL of 1× wash buffer followed by addition of 100 µL secondary antibody-HRP conjugate and incubated for 1 h. Following another wash step, 100 µL of substrate solution was added, and the plate was incubated for 2 to 20 min, during which colour development was monitored. The reaction was terminated by adding 100 µL of stop solution, and absorbance was read at 450 nm.

### Molecular docking of selected phytochemicals against SOD, XO, and caspase-9

The 3D crystal structures of XO, SOD, and caspase-9 were obtained from the Protein Data Bank (PDB) using the IDs 1FIQ, 2C9V, and 2AR9, respectively. Protein structures were visualised and prepared using UCSF Chimera (v1.15), where water molecules and non-essential heteroatoms were removed and polar hydrogens were added prior to docking. The proteins were also prepared accordingly by replacing incomplete side chain and the net charge was computed through antechamber [24, 25]. The prepared proteins were saved in PDBQT format for docking in AutoDock Vina. The grid box and binding pocket size encompassing the active site region of the target protein for XO, SOD and caspase-9 were shown in Table 1. The exhaustiveness was set to 8, and the number of binding modes was set to 10. Protein preparation involved removal of water molecules, addition of polar hydrogens, and assignment of Kollman charges. Metal ions present in SOD were retained during docking. Meanwhile, the ligands, berberine (PubChem ID: 2353), palmatine (PubChem ID: 19009), apigenin (PubChem ID: 5280443), and quercetin (PubChem ID: 5280343) were retrieved from the PubChem database based on their reported antioxidant properties and presence in *T. cordifolia* and *T. crispa*. The active sites of the proteins were identified using the Computed Atlas of Surface Topography of Proteins (CASTp) server. The docking poses and interactions in 3D and 2D were visualised and analysed using Discovery Studio 2021.

**Table 1:** Summary of docking grid parameters for the selected target proteins, showing the grid box centre coordinates (X, Y, Z) and grid dimensions (Å) used to define the active binding regions during molecular docking simulations

Protein (PDB ID)	Coordinates (X, Y,Z)	Grid Size (X, Y,Z) Å
Xanthine Oxidase (1FIQ)	19.035, 46.258, 108.950	(6.716, 6.716, 6.716) Å
Superoxide Dismutase (2C9V)	12.658, -1.051, 10.916	(7.921, 7.921, 7.921) Å
Caspase-9 (2AR9)	10.053, 34.199, 27.109	(6.780, 6.780, 6.780) Å

### Statistical analysis

Data are presented as mean ± standard deviation (SD) from three biological replicates. Statistical analyses were performed using IBM SPSS Statistics version 25 for Windows (NY, USA). Prior to analysis, the data were assessed for normal distribution through Shapiro-Wilk test to ensure the assumptions required for parametric testing were satisfied (*p* > 0.05). Differences among multiple experimental groups were evaluated using one-way analysis of variance (ANOVA), which was selected as it allows comparison of the means across more than two independent groups under the same experimental conditions. When a significant overall effect was observed, Dunnett’s multiple comparison test was performed to compare treatment groups with the positive control (MGO) and negative control (untreated), while Tukey’s post-hoc test was used to evaluate differences among treatment groups. In the graphical representation, * indicates a significant difference compared with the positive control (MGO), ^#^ indicates a significant difference compared with the negative control (untreated cells) and ^Δ^ indicates significant differences among treatment groups within the same species and extract.

## Results and discussion

### MGO induces cellular apoptosis in hFOB 1.19

**Fig. 1:**
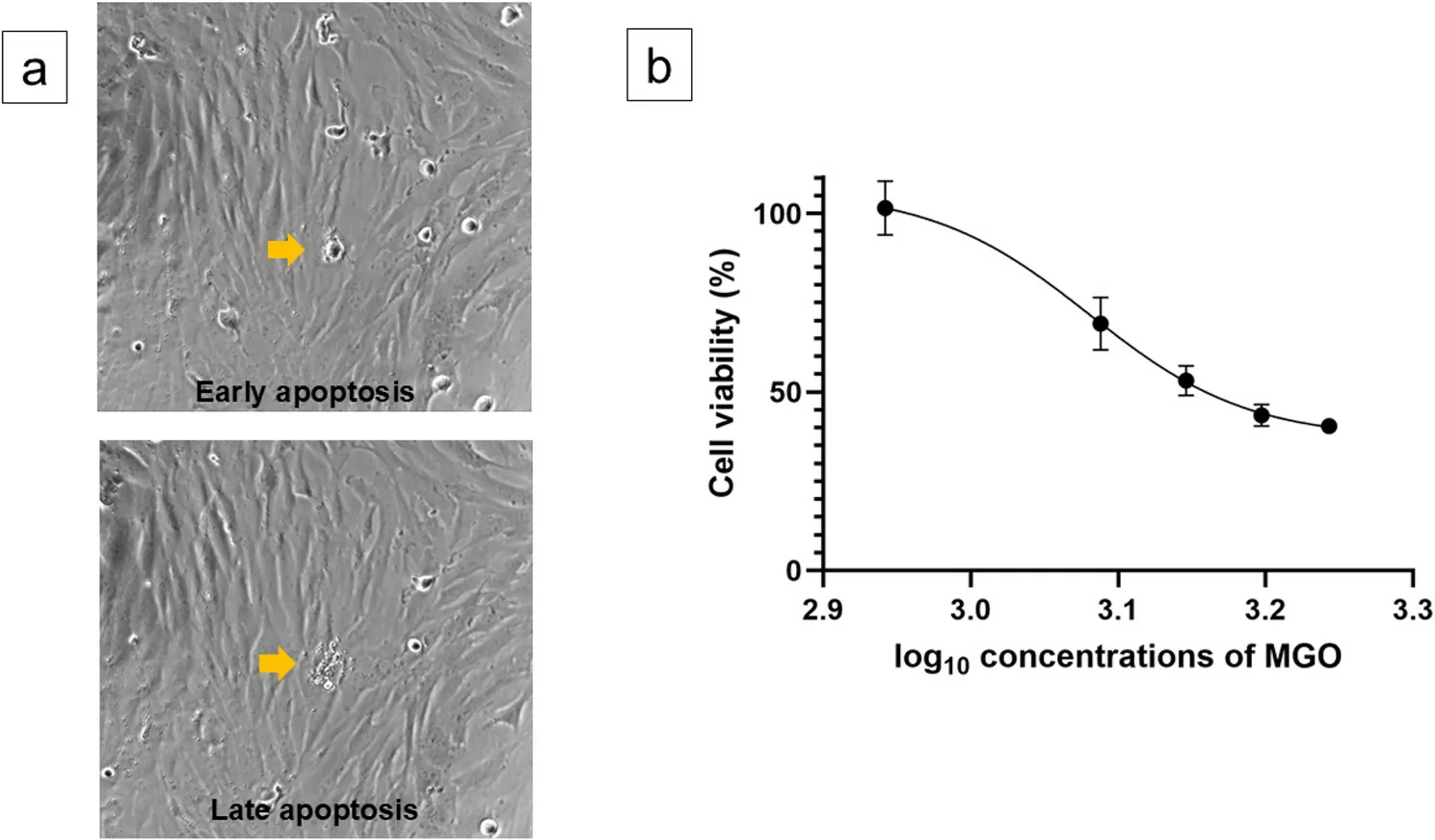
MGO induces hFOB 1.19 apoptosis (**a**) The stimulation of hFOB 1.19 cells with IC_50_ of MGO induced early (cell blebbing) and late stages cell lyses of apoptosis at 100X magnification. **b** IC_50_ determination of MGO in hFOB 1.19 cells indicating the concentration required to reduce cell viability by 50% (*n* = 3)

The microscopy images of early and late apoptosis in MGO-induced hFOB 1.19 cells at the IC_50_ concentration provide visual evidence of the apoptotic effects of MGO as shown in Fig. 1(a). Early apoptotic cells exhibited characteristic morphological changes such as membrane blebbing and chromatin condensation, while late apoptotic cells showed more pronounced features like cellular fragmentation. These observations are consistent with previous studies demonstrating that MGO induces apoptosis through oxidative stress [26]. The cell viability graph further supports this finding, demonstrating a significant reduction in hFOB 1.19 cell viability with increasing MGO concentrations as shown in Fig. 1(b). This dose-response relationship aligns with earlier reports showing that MGO triggers cytotoxicity in osteoblastic cells by generating ROS and inducing mitochondrial dysfunction [27]. Together, these results show the cytotoxic effects of MGO and highlight the need for therapeutic interventions to mitigate its impact on osteoblastic cells.

Furthermore, the IC_50_ value derived from the graph is 1521.1 µM which represents the concentration of MGO required to reduce cell viability by 50%. To date, this is the first report of the IC₅₀ of MGO in hFOB 1.19 cells, providing a reference point for this human osteoblast model. This concentration was subsequently used to stimulate hFOB 1.19 cells in downstream assays. Furthermore, this value serves as a benchmark for evaluating the extent to which antioxidants from *T. crispa* and *T. cordifolia* extracts can protect hFOB 1.19 cells from MGO-induced apoptosis. Previous studies have shown that natural extracts rich in antioxidants, such as those from *Tinospora* species, can scavenge ROS and potentially modulate apoptotic pathways [9, 16]. The current results, combined with evidence from prior studies, provide a strong justification for investigating the protective potential of *T. crispa* and *T. cordifolia* extracts in mitigating MGO-induced cellular damage.

### *T. cordifolia* and *T. crispa* reduced intracellular ROS

MGO was observed to induce a significant increase in intracellular ROS levels, indicating the presence of oxidative stress. MGO glycates proteins to form AGE, with Amadori products as intermediates; this non-enzymatic reaction contributes to increased intracellular ROS production [28].

**Fig. 2:**
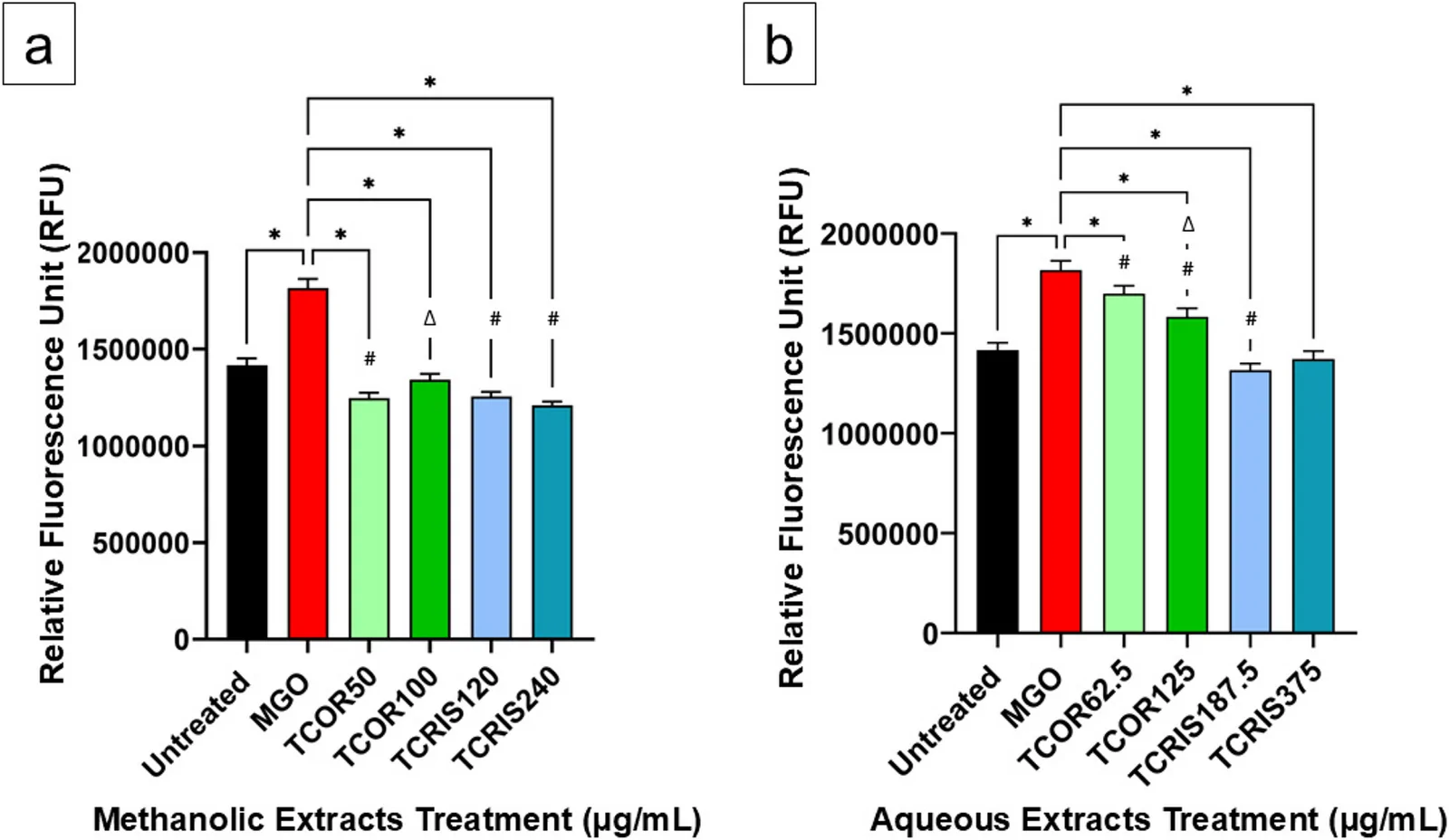
Intracellular ROS expression of MGO-induced hFOB 1.19 cells when (**a**) treated with methanolic extracts of *T. crispa* and *T. cordifolia* and (**b**) aqueous extracts of *T. crispa* and *T. cordifolia*. *p < 0.05 as compared as MGO treated cells (positive control) and ^Δ^*p* < 0.05 as compared to the untreated cells (negative control)

Although the reduction in ROS levels was significant at most tested concentrations, the pattern did not follow a strictly linear dose-response trend (Fig. 2). For the methanolic extracts, *T. cordifolia* at 50 and 100 µg/mL produced a moderate reduction in ROS levels, whereas *T. crispa* at 120 and 240 µg/mL produced slightly stronger effects. However, the difference at the highest concentrations was minimal Fig. 2(a). The aqueous extracts exhibited a similar non-linear pattern, with *T. cordifolia* at 62.5 µg/mL showing a limited effect, while higher concentrations of *T. cordifolia* (125 µg/mL) and *T. crispa* (187.5 to 375 µg/mL) had notably reduced ROS levels compared to the MGO-induced group Fig. 2(b). The methanolic extracts consistently showed stronger antioxidant effects than the aqueous extracts across the tested concentrations, suggesting a higher efficiency in extracting both polar and moderately non-polar bioactive compounds. These include flavonoids (e.g., quercetin, apigenin), phenolic acids, and alkaloids (e.g., berberine, palmatine) [15, 29]. In contrast, water, being highly polar, primarily extracts water-soluble compounds, thereby limiting the extractions towards high polarity compounds [30].

The findings are supported by previous studies reporting that the extracts possess free radical scavenging activity as demonstrated by DPPH antioxidant assay [9, 16]. The DPPH assay evaluates the capacity of these extracts to donate electrons or hydrogen atoms to neutralise free radicals, which is a key characteristic of antioxidant compounds. Such electron-donating properties may enable the bioactive constituents of these extracts to directly quench intracellular ROS generated during oxidative stress. Similar observations have been reported where plant extracts exhibiting strong DPPH radical scavenging activity also supressed intracellular ROS production in a cell-based model [31]. In bone cells, excessive ROS production induced by MGO can impair cellular function and contribute to oxidative damage. Excessive ROS accumulation in osteoblast can reduce their viability and promote apoptosis, ultimately compromising bone formation [32, 33]. While the DPPH assay reflects the chemical radical-scavenging capacity of a compound in a cell-free system, the current findings suggest that these antioxidant properties may also translate into a biological context, contributing to the mitigation of oxidative stress at the cellular level. Therefore, the ROS-lowering effect observed in this study indicate that *T. crispa* and *T. cordifolia* can exert antioxidant activity within a biological system.

### *T*. *cordifolia* and *T*. *crispa* reduced caspase-3 expression 

**Fig. 3:**
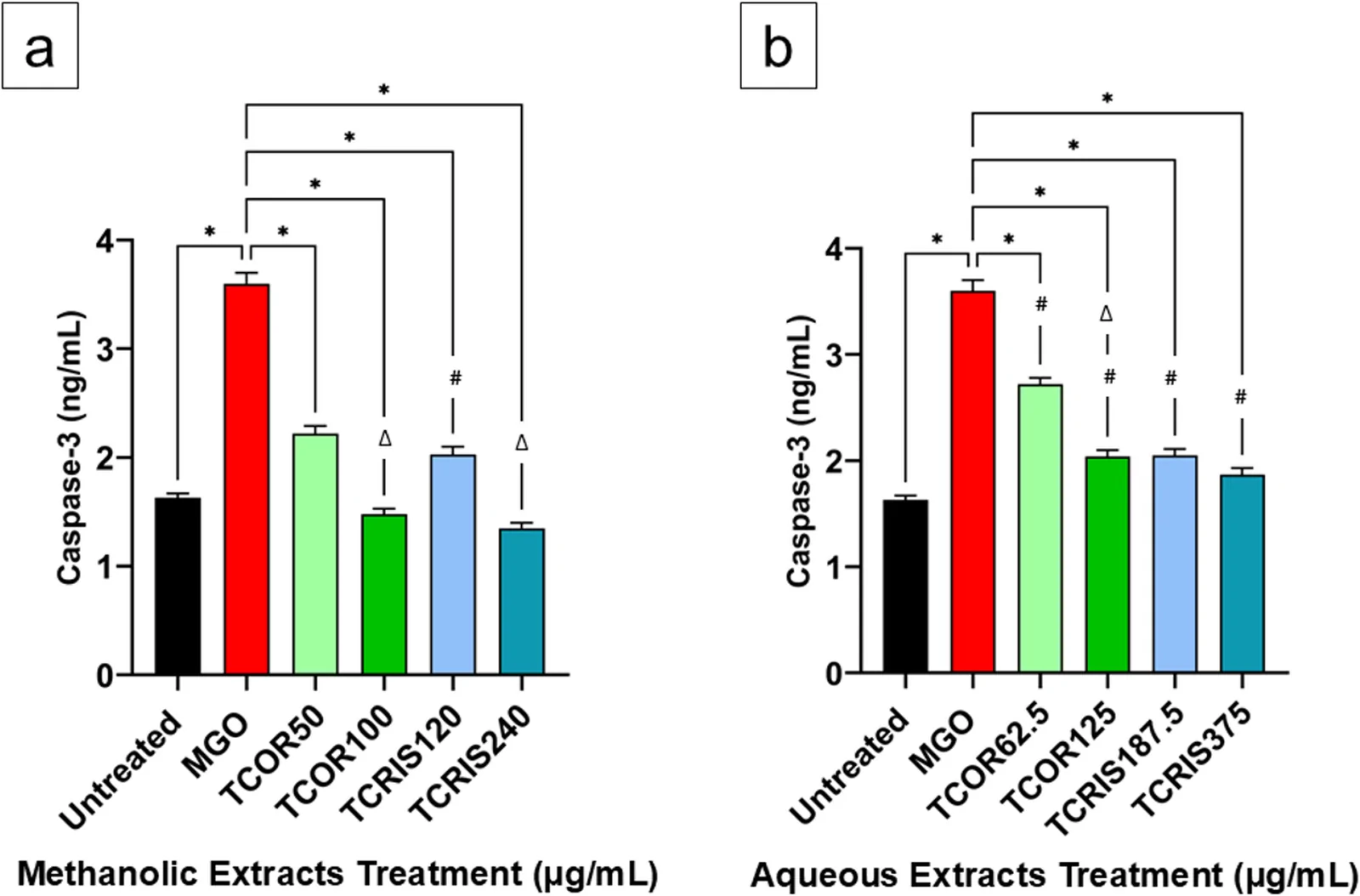
Caspase-3 expression of MGO-induced hFOB 1.19 cells when treated with (**a**) methanolic extracts of *T. crispa* and *T. cordifolia* and (**b**) aqueous extracts of *T. crispa* and *T. cordifolia*. *p < 0.05 as compared as MGO treated cells (positive control) and ^Δ^*p* < 0.05 as compared to the untreated cells (negative control)

Caspase-3 is a key executioner protease in the apoptotic pathway. In this study, stimulation with MGO significantly upregulated caspase-3 expression in hFOB 1.19 (*p* < 0.05) by 54.72%, indicating that MGO-induced oxidative stress may have triggered apoptosis in osteoblastic cells as shown in Fig. 3. Previous studies have similarly reported that MGO promotes apoptotic signalling through increased caspase-3 expression in various cell types, including MCF-7 and MC3T3-E1 cells [34, 35].

Treatment with the crude methanolic extracts significantly reduced caspase-3 expression in MGO-induced cells (*p* < 0.05), by approximately 38.3 to 62.5% compared with the MGO control shown in Fig. 3(a). Similarly, the crude aqueous extracts also reduced caspase-3 expression by 24.4 to 48.1%, as shown in Fig. 3(b). This downregulation of caspase-3 expression suggests that the extracts may modulate apoptosis-related signalling pathways. Although multiple concentrations of the extracts were tested, the reductions in caspase-3 expression did not follow a strictly concentration-dependent pattern, suggesting that the anti-apoptotic effect may occur across a relatively broad effective concentration range rather than exhibiting a clear linear dose-response relationship.

Notably, this reduction in caspase-3 expression is consistent with the earlier observation that both extracts significantly lowered intracellular ROS levels in MGO-induced cells. Excessive ROS accumulation is known to trigger apoptotic signalling pathways in osteoblasts; the observed suppression of ROS may have contributed to the attenuation of caspase-3 expression [36]. These findings suggest that the antioxidant properties of *T. crispa* and *T. cordifolia* may help mitigate oxidative stress-induced apoptosis, thereby preserving osteoblasts’ viability under MGO-induced oxidative conditions.

### *T. cordifolia* and *T. crispa* reduced AGE expression

**Fig. 4:**
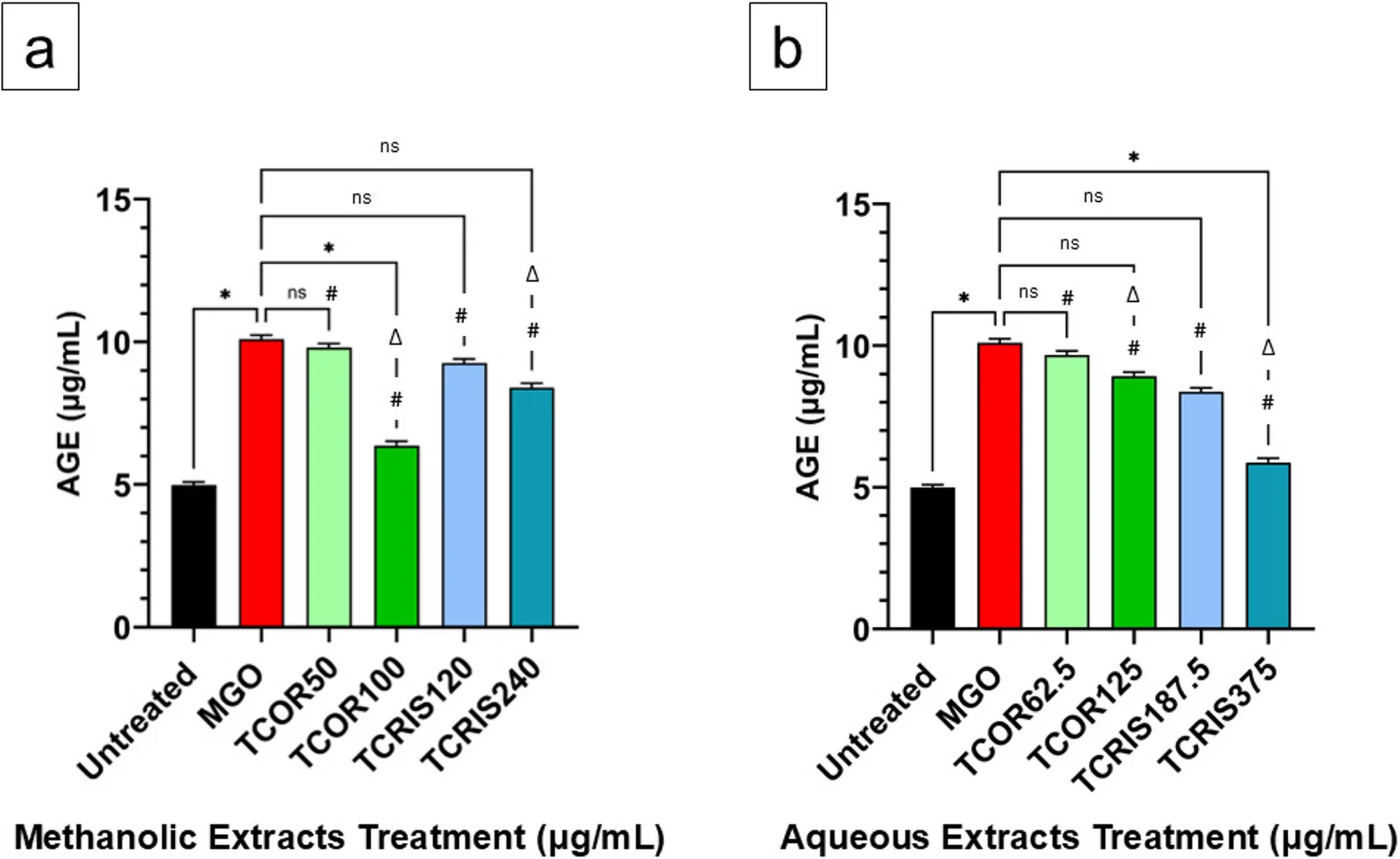
AGE expression of MGO-induced hFOB 1.19 cells when treated with (**a**) methanolic extracts of *T. crispa* and *T. cordifolia* and (**b**) aqueous extracts of *T. crispa* and *T. cordifolia*. *p < 0.05 as compared as MGO treated cells (positive control) and ^Δ^*p* < 0.05 as compared to the untreated cells (negative control)

In contrast to the marked reductions observed in intracellular ROS and caspase-3 expression, the extracts produced only limited effects on AGE expression. Among the treatments tested, only the methanolic extract of *T. cordifolia* at 100 µg/mL and the aqueous extract of *T. crispa* at 375 µg/mL significantly reduced AGE expression, as shown in Fig. 4(a, b). This selective effect may reflect differences in the bioactive constituents present in specific extracts as reported previously [9], which could influence their ability to interfere with glycation pathways. Although the extracts effectively modulated oxidative stress and apoptosis-related processes, AGE formation primarily occurs through non-enzymatic glycation reactions involving reactive carbonyl species including MGO. These glycation reactions proceed through biochemical pathways that are distinct from those regulating ROS production and apoptosis. In addition, AGE formation is generally a slower and more progressive process compared with oxidative stress responses [37], which may explain the comparatively modest reduction observed in the present study. Therefore, while *T. cordifolia* and *T. crispa* appear to effectively target oxidative stress-related mechanisms, further investigations focusing on compounds with stronger anti-glycation properties may be required to more effectively inhibit AGE accumulation.

### Molecular docking analysis of selected bioactive compounds from *T. cordifolia *and *T. crispa*

Molecular docking was performed using selected bioactive compounds previously reported in *Tinospora* species, including berberine and palmatine, which are well-known alkaloids associated with antioxidant and anti-inflammatory activities [38–41]. In addition, the flavonoids apigenin and quercetin were included due to their reported antioxidant and anti-glycation properties [42, 43]. These compounds were selected as representative phytochemicals based on previous phytochemical and pharmacological studies of *Tinospora* species. The docking analysis was therefore conducted to explore potential molecular interactions between these reported bioactive constituents and the selected target proteins.

**Fig. 5:**
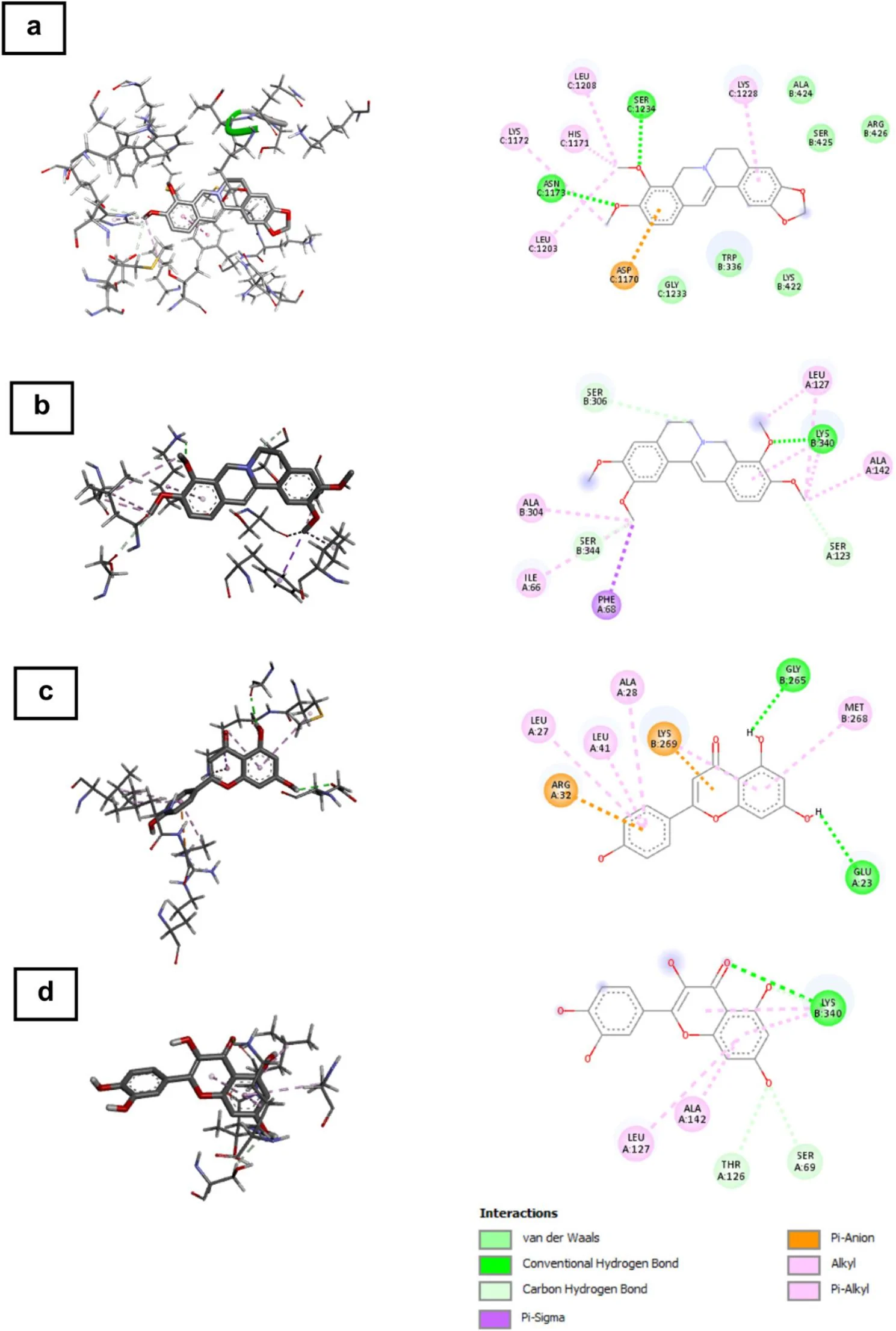
Predicted binding modes of selected phytochemicals within the active site of xanthine oxidase (XO). The left panels show the three-dimensional (3D) protein–ligand interaction models, illustrating the orientation of each ligand within the XO binding pocket and its spatial relationship with surrounding amino acid residues. The right panels present the corresponding two-dimensional (2D) interaction maps, identifying the specific residues involved in ligand stabilization through hydrogen bonding, π–anion, π–sigma, alkyl, π–alkyl, and van der Waals interactions. The docked compounds shown are (**a**) berberine, (**b**) palmatine, (**c**) apigenin, and (**d**) quercetin

Molecular docking was performed to examine the interaction between the ligands and XO as shown in Fig. 5. Each ligand was docked with XO using Autodock Vina and the binding energies of these poses are presented in Table 2. Binding energy represents the amount of energy released when the ligand binds to the protein, with more negative values indicating greater stability of the ligand-complex [44]. XO is a key enzyme involved in purine metabolism, catalysing the oxidation of hypoxanthine to xanthine and subsequently the production of uric acid. During this process, XO generates significant amounts of ROS. Excessive ROS production can lead to oxidative stress, which is implicated in various pathological conditions, including bone dysregulation [6]. Therefore, modulating XO represents an option for investigating the reduction of oxidative damage. In silico analysis revealed that berberine exhibited the highest binding affinity (-8.3 kcal/mol), followed by palmatine (-7.9 kcal/mol), apigenin (-7.8 kcal/mol), and quercetin (-7.6 kcal/mol). These in silico results suggest that berberine interacts with XO binding site, indicating a structural mechanism for potential enzyme inhibition and subsequent reduction in ROS generation and downstream oxidative stress. These findings are consistent with previous studies that identified dihydroberberine, a reduced form of berberine, as a potent XO inhibitor capable of mitigating ROS generation [45, 46]. Apigenin has also been reported to exhibit a strong affinity for XO, with a docking score of -8.7 kcal/mol [47]. Overall, the strong binding affinity and favourable interactions highlight these bioactive compounds as potential candidates in modulating XO activities. Modulating this pathway could represent an approach for addressing oxidative stress-related disorders and investigating downstream effects on bone turnover.

**Table 2:** The molecular docking score of XO with bioactive compounds

Protein (PDB ID)	Bioactive compound (PubChem ID)	Docking Score (kcal/mol)	Residue(s) involved	Bonds
Xanthine oxidase (XO)	Berberine (2353)	-8.3	Ser123,Ser117	Conventional hydrogen bond
			Gly123,Trp336,Lys422	Carbon hydrogen bonds
			Ala424, Ser425	Van der Waals
			Asp117	Π-anion
			His117,Lys122, Leu120, Lys117	Π-alkyl and alkyl bonds
	Palmatine (19009)	-7.9	Lys340	Conventional hydrogen bond
			Ser334, Ser306, Ser123	Carbon hydrogen bond
			Phe68	Π-sigma
			Lys340, Leu127, Ala142, Ile66, Ala304	Π-alkyl and alkyl bonds
	Apigenin (5280433)	-7.8	Gly265, Glu23	Conventional hydrogen bond
			Lys269, Arg32	Π-cation
			Ala27, Leu41, Leu27	Π-alkyl
	Quercetin (5280343)	-7.6	Lys340	Conventional hydrogen bond
			Ser69, Thr126	Carbon hydrogen bond
			Ala142, Leu122	Π-alkyl

Meanwhile, SOD plays a critical role in cellular defence against oxidative stress by catalysing the dismutation of superoxide radicals into oxygen and hydrogen peroxide, thereby reducing ROS levels [48]. In MGO-induced hFOB 1.19 cells, the overproduction of ROS and the formation of AGEs are key contributors to cellular damage and apoptosis. Molecular docking studies targeting SOD revealed that quercetin exhibited the highest binding affinity (-7.9 kcal/mol), followed by apigenin (-7.8 kcal/mol), palmatine (-7.4 kcal/mol) and berberine (-7.4 kcal/mol) as shown in Table 3. This strong interaction suggests that these bioactive compounds may modulate SOD stability, potentially improving the enzyme’s ability to neutralize superoxide radicals. The ability of the extracts in modulating SOD may help mitigate MGO-induced oxidative stress, potentially reducing ROS accumulation and suppressing AGE formation. For example, literature indicates that quercetin modulates glutathione (GSH) levels and may subsequently regulate SOD [49].

**Table 3:** The molecular docking score of SOD with bioactive compounds

Protein (PDB ID)	Bioactive compound (PubChem ID)	Docking Score (kcal/mol)	Residue(s) involved	Bonds
Superoxide dismutase, SOD (2C9V)	Berberine (2353)	-7.4	Asn53	Conventional hydrogen bond
			Val148, Lys9	Π-alkyl and alkyl bonds
	Palmatine (19009)	-7.4	Val(F)148, Val(A)148	Conventional hydrogen bond
			Thr54	Carbon hydrogen bond
			Lys9, Val7	Π-alkyl and alkyl bonds
	Apigenin (5280433)	-7.8	Gly56, Val7	Conventional hydrogen bond
			Gly147	Carbon hydrogen bond
			Val148, Val7	Π-alkyl
	Quercetin (5280343)	-7.9	Asn53, Val(F)148, Asp11	Conventional hydrogen bond
			Val(A)148	Carbon hydrogen bond
			Lys9	Π-alkyl

In addition, MGO not only promotes ROS generation but also facilitate the formation of AGE that impair cellular function and trigger apoptosis [50]. By targeting SOD, quercetin and other high-affinity compounds may disrupt this vicious cycle, thereby preserving cellular integrity and function. Previous studies have shown that SOD upregulation can attenuate oxidative stress and AGE accumulation in various cell models [51].

**Fig. 6:**
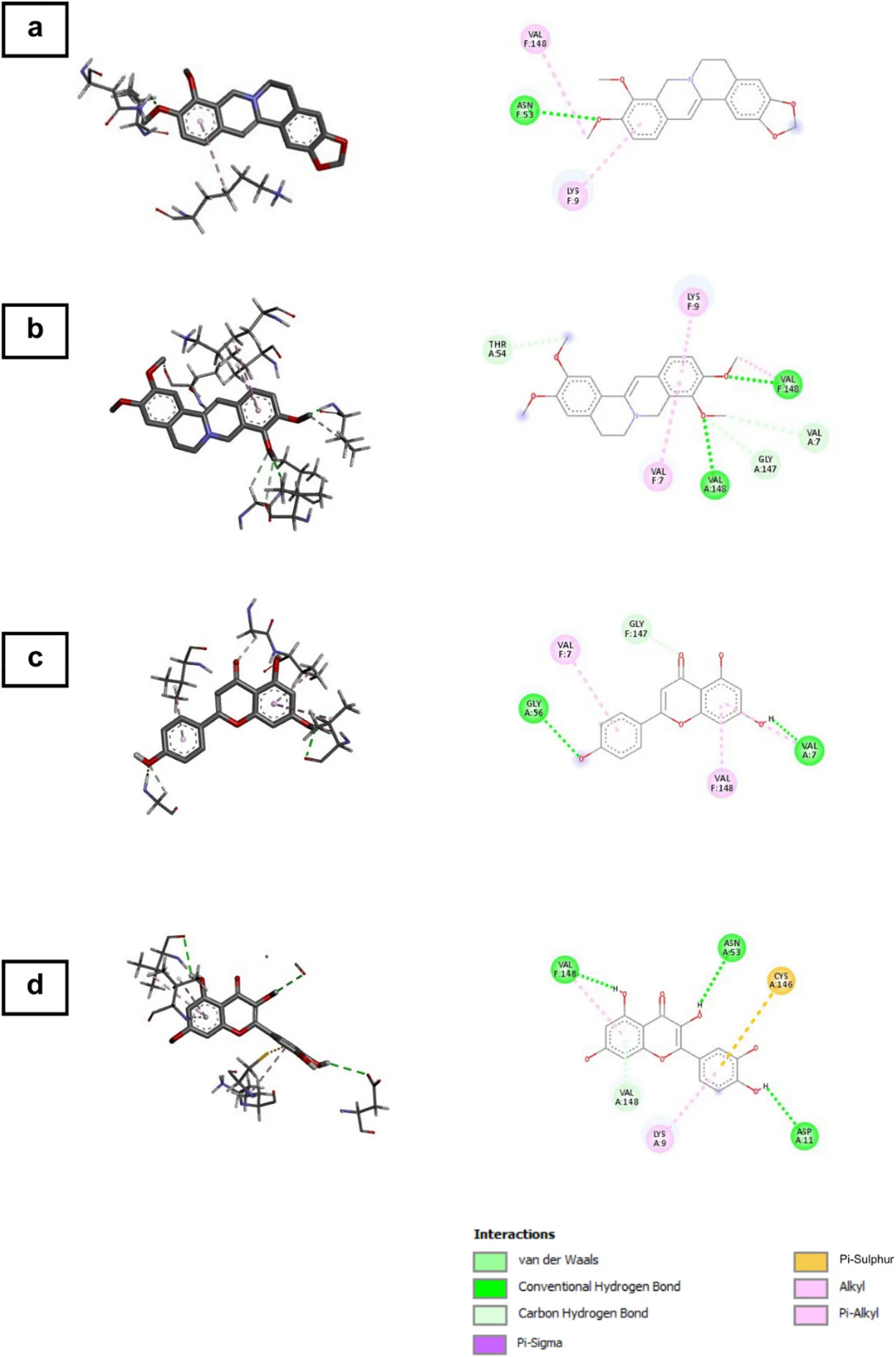
Predicted binding modes of selected phytochemicals within the active site of superoxide dismutase (SOD). The left panels illustrate the three-dimensional (3D) protein–ligand interaction models, showing the orientation of each compound within the SOD binding pocket and its spatial proximity to surrounding amino acid residues. The right panels show the corresponding two-dimensional (2D) interaction maps, highlighting the residues involved in ligand stabilization through conventional hydrogen bonding, carbon hydrogen bonding, π–sulphur, π–sigma, alkyl, π–alkyl, and van der Waals interactions. The docked compounds are (**a**) berberine, (**b**) palmatine, (**c**) apigenin, and (**d**) quercetin

Furthermore, the molecular docking analysis of SOD revealed strong interactions between the bioactive compounds from *T. crispa* and *T. cordifolia* extracts and the enzyme’s active site as shown in Fig. 6. The 2D interaction diagram illustrates the key molecular interactions stabilizing these ligand-protein complexes, including conventional hydrogen bonding, π-π stacking, and van der Waals forces. Notably, quercetin formed hydrogen bonds with Asn53 and Asp11, as well as π-alkyl interactions with key hydrophobic residues, enhancing its stability within the active site. The favourable binding profiles of these compounds suggest they may play a role in supporting SOD-mediated antioxidant defences. This mechanism could ultimately assist in neutralizing superoxide radicals and mitigating oxidative stress in osteoblast cells. However, it is also important to note that this interaction requires more enzymatic validation to assess its full potential. These findings support the antioxidant role of *T. crispa* and *T. cordifolia* extracts, further emphasizing their potential therapeutic application in combating oxidative stress-induced damage in bone cells.

The strong binding affinities observed in the molecular docking of caspase-9 with bioactive compounds from *T. crispa* and *T. cordifolia* extracts suggest that these compounds may interfere with caspase-9 as shown in Table 4. Caspase-9, an initiator caspase in the intrinsic apoptotic pathway, is responsible for activating downstream executioner caspases such as caspase-3 [52]. Among the tested ligands, berberine exhibited the highest binding affinity (-8.7 kcal/mol), followed by palmatine (-8.0 kcal/mol), apigenin (-7.7 kcal/mol), and quercetin (-7.6 kcal/mol). The docking results indicate that these bioactive compounds may modulate caspase-9, thereby potentially preventing its activation and subsequent propagation of the apoptotic cascade. In silico suggests that berberine may potentially interact with caspase-9 active site, representing a potential structural mechanism for the previously observed reduction of caspase-3 in MGO-induced hFOB 1.19 cells.

**Table 4:** The molecular docking score of caspase-9 with bioactive compounds

Protein (PDB ID)	Bioactive compound (PubChem ID)	Docking Score (kcal/mol)	Residue(s) involved	Bonds
Caspase-9 (2AR9)	Berberine (2353)	-8.7	Lys278, Pro279, Asp228	Conventional hydrogen bond
			Tyr153, Lys278, Pro279, Ile154, Ala149	Π-alkyl and alkyl bonds
	Palmatine (19009)	-8.0	Lys278	Π-anion
			Glu143, Gly276	Carbon hydrogen bond
			Ile(C)154, Ile(A)154, Ala149	Π-alkyl and alkyl bonds
	Apigenin (5280433)	-7.7	Ile154, Asp228	Conventional hydrogen bond
			Lys409, Lys278	Π-alkyl and alkyl
			Ala149	Π-alkyl
	Quercetin (5280343)	-7.6	Asp228, Ser(A)156, Ser(C)156, Gly227	Conventional hydrogen bond
			Ile154	Π-Sigma bond
			Ala149, Ile154	Π-alkyl and alkyl

The reduction in caspase-3 expression observed in the in vitro ELISA assay further supports the anti-apoptotic effects of *T. crispa* and *T. cordifolia* extracts. Caspase-3 is the key executioner caspase responsible for the final stages of apoptosis, and its downregulation indicates a protective effect against MGO-induced oxidative stress [53]. MGO is known to trigger apoptosis through the intrinsic pathway, where oxidative stress and mitochondrial dysfunction lead to the release of cytochrome-c, followed by activation of caspase-9 and the downstream activation of caspase-3 [53, 54]. Therefore, the observed decrease in caspase-3 expression may be associated with upstream modulation of caspase-9 as well as the antioxidant properties of the extracts. Bioactive constituents present in *T. crispa* and *T. cordifolia* have been reported to possess antioxidant capacity [9] and may help preserve mitochondrial integrity, thereby preventing cytochrome-c release and subsequent activation of the caspase cascade. Collectively, these findings suggest that the extracts exert protective effects against MGO-induced apoptosis through modulation of mitochondrial apoptotic signalling in osteoblast cells.

**Fig. 7:**
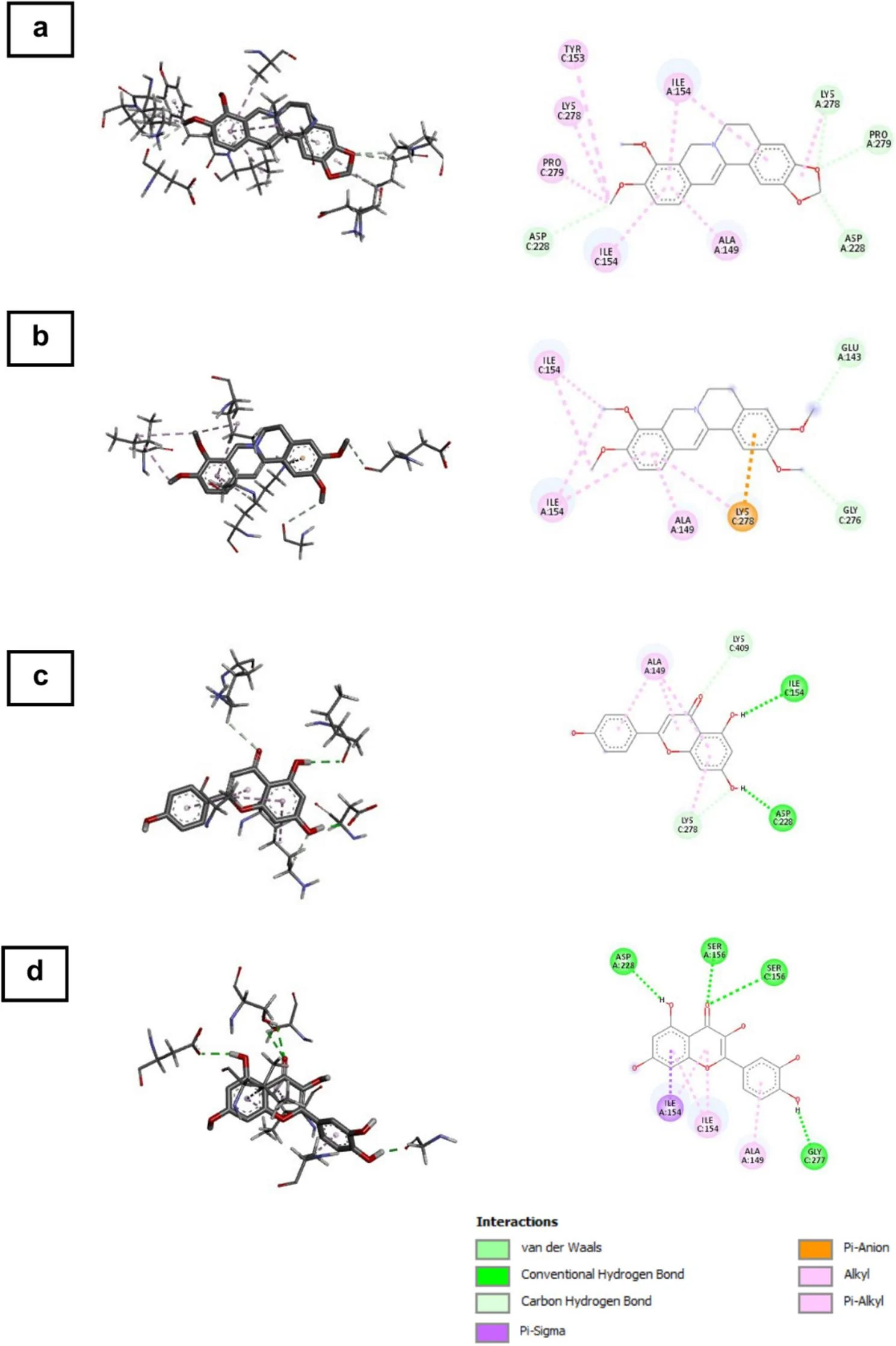
Predicted binding modes of selected phytochemicals within the active site of caspase-9. The left panels illustrate the three-dimensional (3D) protein–ligand interaction models, showing the orientation of each compound within the SOD binding pocket and its spatial proximity to surrounding amino acid residues. The right panels show the corresponding two-dimensional (2D) interaction maps, highlighting the residues involved in ligand stabilization through conventional hydrogen bonding, carbon hydrogen bonding, π–sulphur, π–sigma, alkyl, π–alkyl, and van der Waals interactions. The docked compounds are (**a**) berberine, (**b**) palmatine, (**c**) apigenin, and (**d**) quercetin

The 2D molecular docking analysis of caspase-9 offers insights into how bioactive compounds from *T. crispa* and *T. cordifolia* may interact to inhibit the apoptotic pathway, as shown in Fig. 7. The 2D interaction diagram highlights key molecular interactions, including hydrogen bonding, π-alkyl interaction, and van der Waals forces, that contribute to the stability of the ligand-protein complexes. Notably, berberine formed strong hydrogen bonds with Asp228 and Pro279, as well as π-alkyl interactions with key hydrophobic residues such as Ile154 and Ala149, reinforcing its binding within the active site of caspase-9.

## Conclusion

This study highlights the potential of *T. crispa* and *T. cordifolia* extracts in modulating markers of oxidative stress and apoptosis in hFOB 1.19 osteoblastic cells. The significant reduction in intracellular ROS and caspase-3 expression following treatment with both methanolic and aqueous extracts suggests potential antioxidant and anti-apoptotic properties. However, the limited reduction in AGE expression suggests that while the extracts modulate oxidative stress and apoptosis markers, they may not directly alter the glycation process or AGE formation. This emphasises the need for further investigation into the specific mechanisms by which these extracts influence glycation pathways and AGE accumulation.

As observed in this study, the protective effect of the extracts may be due to their bioactive compounds, such as berberine, palmatine, apigenin, and quercetin, which demonstrated strong binding affinities with key enzymes including XO, SOD, and caspase-9 in molecular docking simulations. Through these predicted interactions and observed changes in expression, the extracts may influence the oxidative stress-apoptosis cascade, thereby supporting cellular integrity and function. However, while these in silico findings offer valuable predictive insights into the ligand-protein interactions of *Tinospora* compounds, future enzymatic and cellular assays are warranted to validate these computational predictions and definitively confirm their osteoprotective mechanisms.

In conclusion, *T. crispa* and *T. cordifolia* extracts exhibit promising potential in protecting osteoblasts from MGO-induced oxidative stress and apoptotic markers. The molecular docking results provide insights into the potential interactions of the bioactive compounds, particularly regarding their binding capacity toward XO and SOD, which warrants further validation as potential targets for managing oxidative stress-related bone disorders. Future studies should focus on isolating and characterizing the specific bioactive compounds responsible for these changes, evaluating their efficacy in vivo, and exploring potential synergistic effects. Additionally, further research is needed to clarify the extracts’ impact on AGE expression and glycation pathways to provide a more comprehensive understanding of anti-glycation bioactivity.

## Supplementary Information

Supplementary Material 1.

## Data Availability

The datasets used and/or analysed during the current study are available from the corresponding author on reasonable request.

## Abbreviations

AGE: Advanced glycation end products
ELISA: Enzyme-linked immunosorbent assay
MGO: Methylglyoxal
PDB: Protein Data Bank
ROS: Reactive oxygen species
SOD: Superoxide dismutase
*T. cordifolia*: *Tinospora cordifolia*
*T. crispa*: *Tinospora crispa*
UCSF: University of California, San Francisco
XO: Xanthine oxidase
