# Supplementary material for: Protective effects of Tinospora crispa and Tinospora cordifolia extracts against methylglyoxal-induced oxidative stress and apoptosis in hFOB 1.19 cells: ELISA-based evaluation and molecular docking insights with antioxidant enzymes
Source: BMC Complement Med Ther. 2026 Jun 24;26:266. doi: 10.1186/s12906-026-05450-y (PMC13551757; doi:10.1186/s12906-026-05450-y)

Supplementary Figure 1 – MGO-stimulated hFOB1.19 Viability and IC50 of MGO

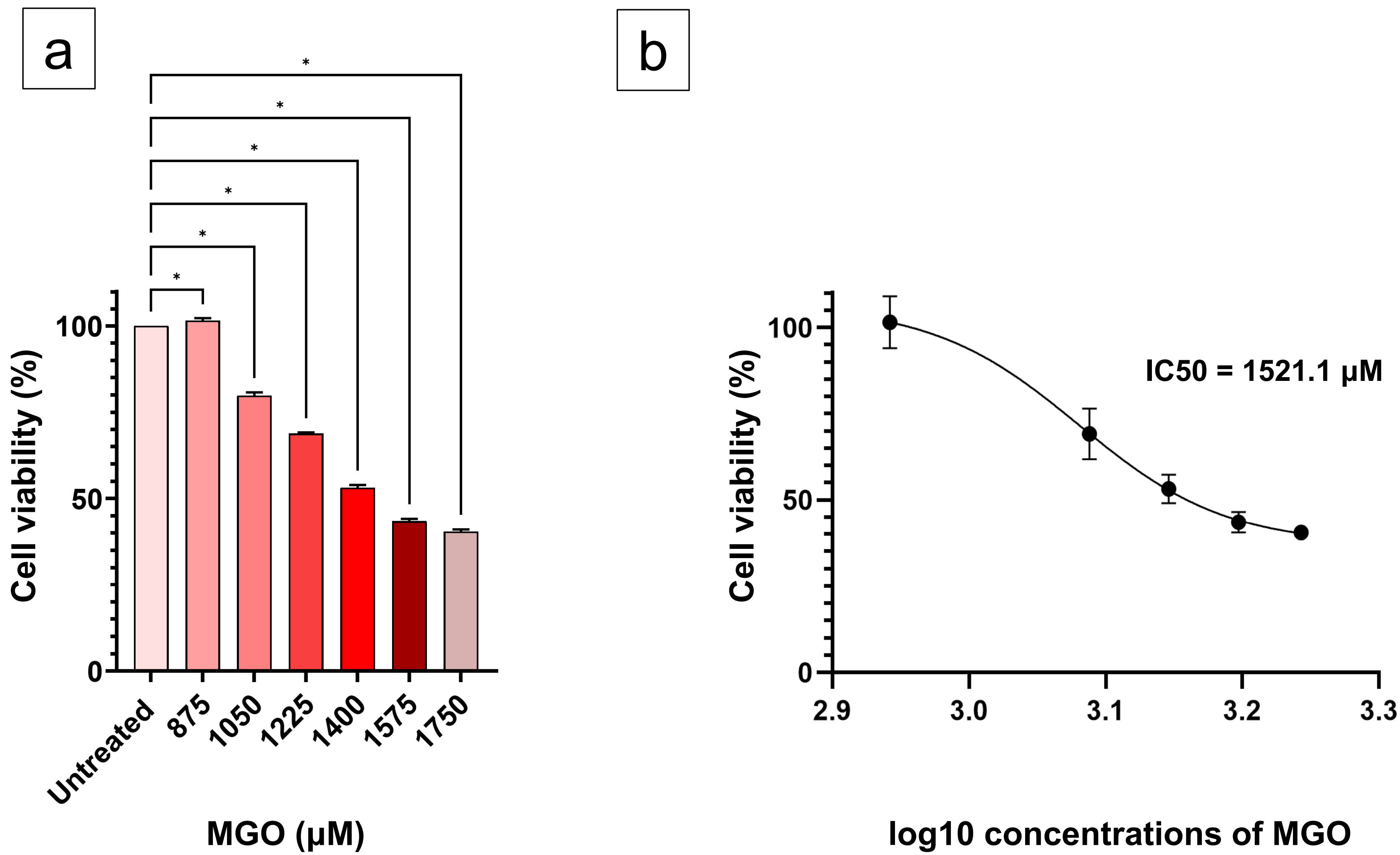

Supplementary Figure 2 – Methanolic and Aqueous *T. cordifolia* on hFOB1.19 Viability

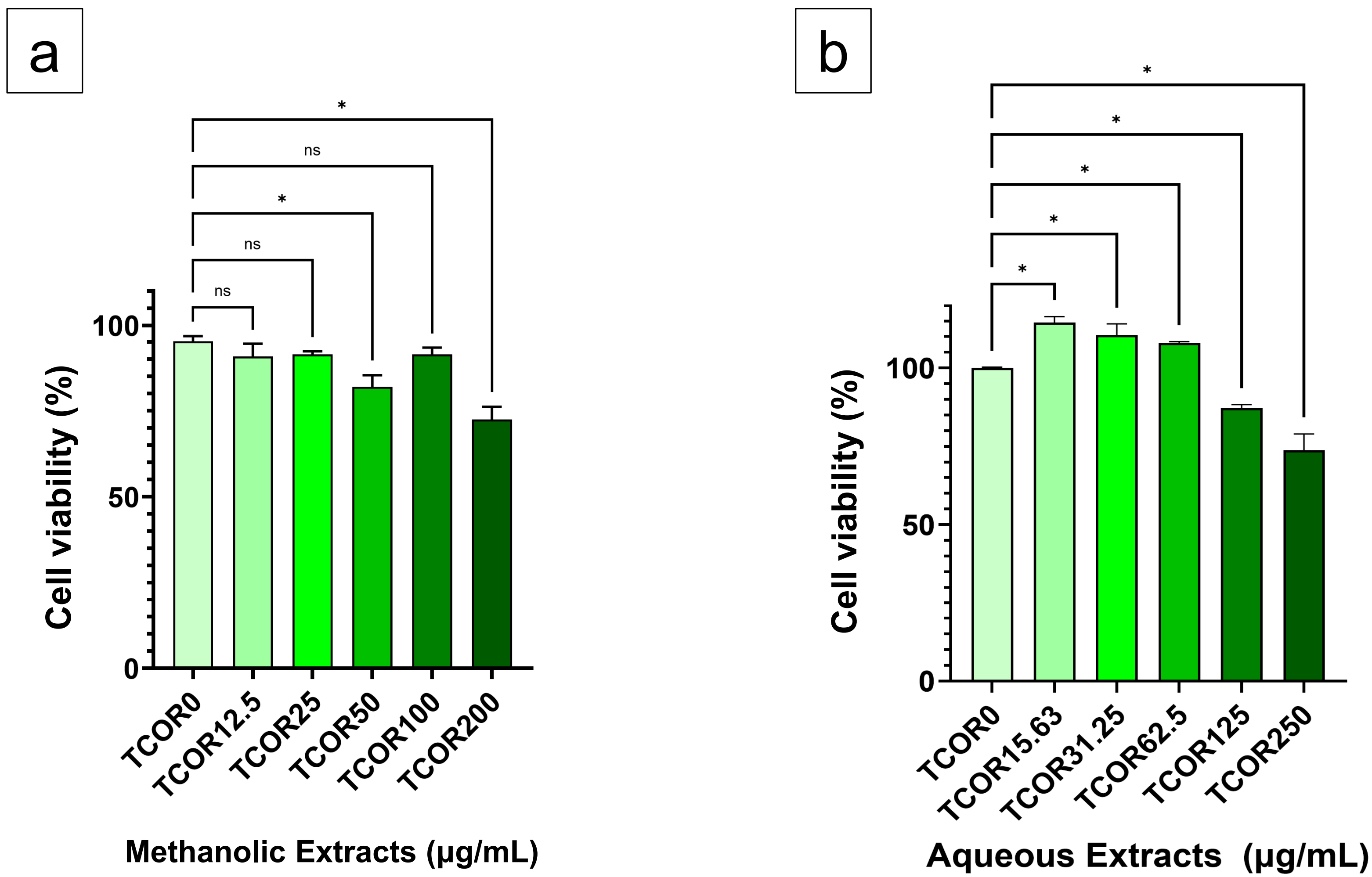

Supplementary Figure 3 – Methanolic and Aqueous *T. crispa* on hFOB1.19 Viability

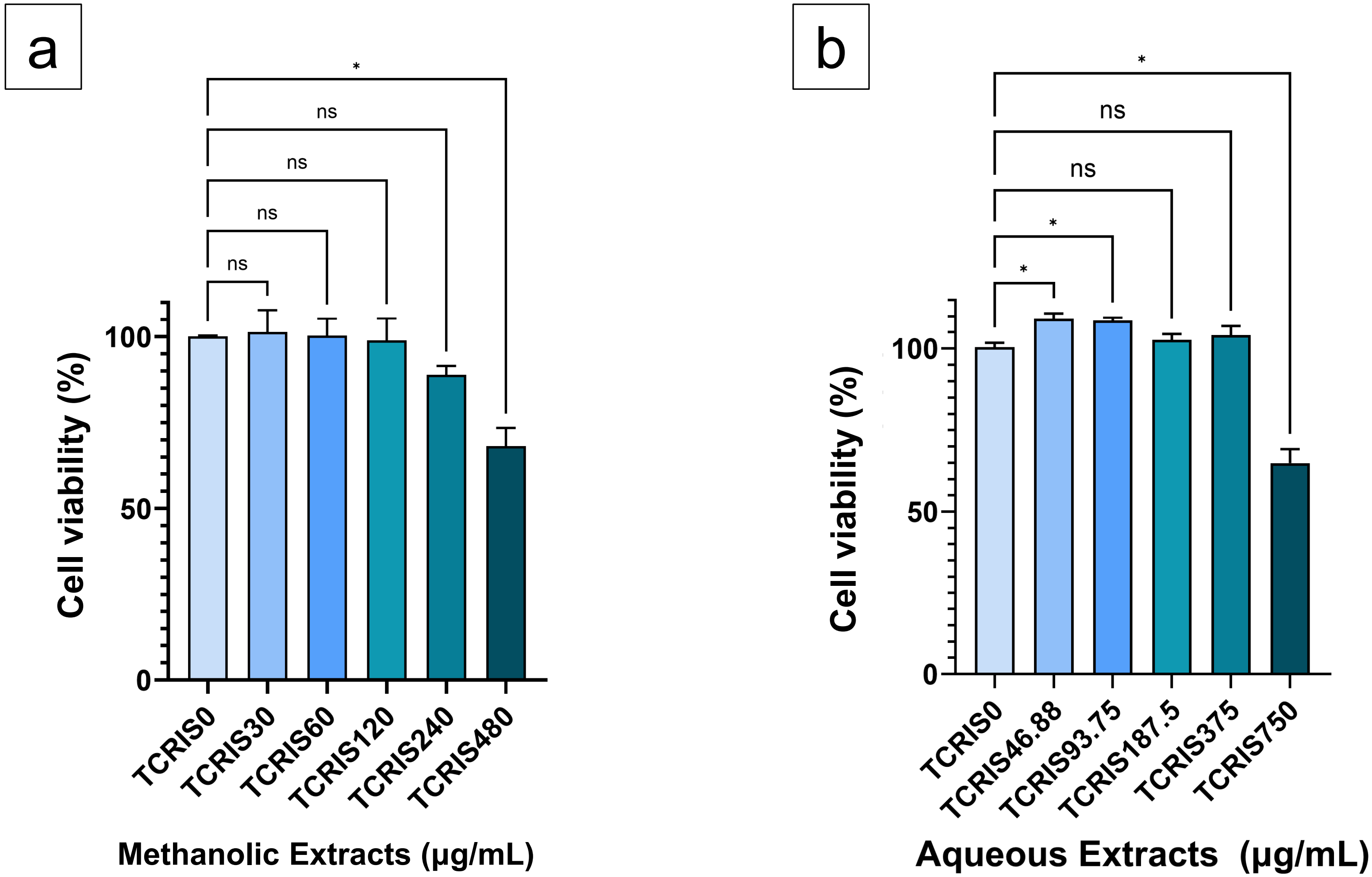

Supplementary Figure 4 – Molecular Docking Study Design

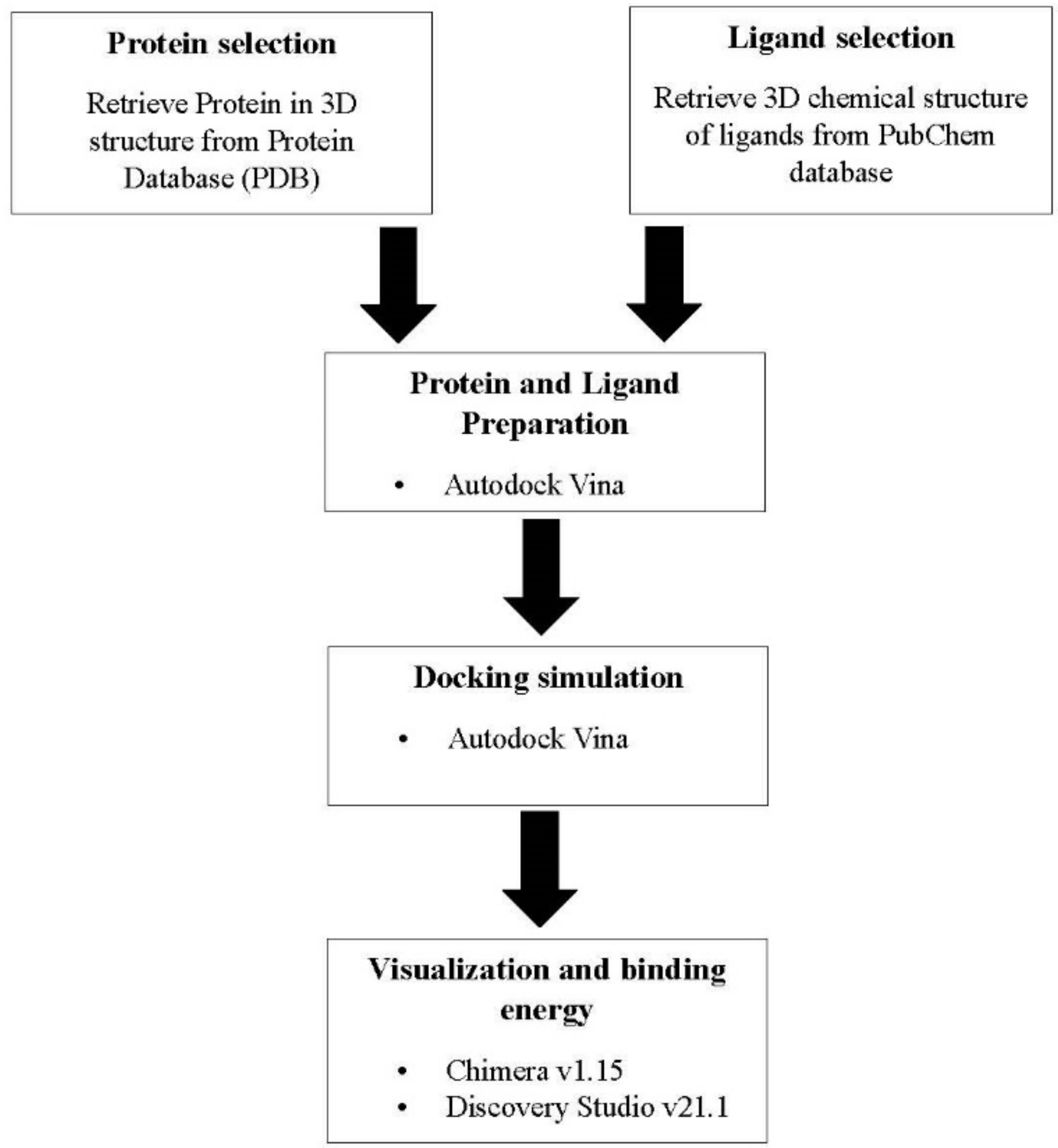

Supplement: Supplementary file 1 [file 12906_2026_5450_MOESM1_ESM.pdf]
